# Morphological and hand grip strength characteristics and differences between participants of the 2022 world rowing championship

**DOI:** 10.3389/fspor.2023.1115336

**Published:** 2023-03-09

**Authors:** Jan Busta, Jaroslav Hellebrand, Ivana Kinkorová, Tomáš Macas

**Affiliations:** Department of Swimming, Water and Technical Sports, Faculty of Physical Education and Sport, Charles University in Prague, Prague, Czech Republic

**Keywords:** rowing, anthropometry, somatotype and body composition, elite sport, female sport, body constitution

## Abstract

**Introduction:**

Rowing is a strength endurance type of sport, and morphology and mass are undoubtedly performance-related factors. Precisely identifying these morphological factors associated with performance, can assist the exercise scientists and coaches in selecting and developing talented athletes. There is however, a lack of anthropometric data collected at either World Championship or Olympic Games level. The aims of this study were to describe and compare the morphology and basic strength characteristic of male and female heavyweight and lightweight rowers competing at 2022 World Championship (18.–25. September, Račice, Czech Republic).

**Methods:**

A total of 68 athletes (of 46 male competitors: 15 competed in the lightweight category and 31 in the heavyweight category; of 22 female athletes: 6 competed in the lightweight category and 16 in the heavyweight category) were assessed using anthropometric methods, bioimpedance analysis and performed a hand-grip test.

**Results:**

Between heavyweight and lightweight male rowers there were a statistically and practically significant differences in all monitored aspects except the sport age, sitting height/body height ratio and arm span/body height ratio. Between heavyweight and lightweight female rowers there were also statistically and practically significant differences in all monitored aspects except the identical indicators as in male.

**Discussion:**

Within this research it can be argued that female rowers are in many anthropometric aspects more similar to their male counterparts than to female rowers in the lightweight category. In some anthropometric aspects (BMI, thigh girth, calf girth), female rowers are even more similar to male heavyweight than to male lightweight rowers. The physical characteristics of elite male and female lightweight rowers differ radically from those of heavyweight. From a practical point of view, this research can be used to determine what type of athletes should be recruited or selected for heavy category and what type for lightweight category in male and female rowing based on the somatotype.

## Introduction

Rowing is a strength endurance type of sport, and morphology and mass are undoubtedly performance-related factors (1). Body height and mass strongly affects the career in men (2). The studies of the morphology of elite male and female rowers are very useful in view of the rapid evolution of sports and sportspeople (3). The best athletes had higher values of segmental lengths, circumferences, and muscle widths (4). Body height (*r* = −0.801), body mass (*r* = −0.812), arm span (*r* = −0.715), leg length (*r* = −0.703), and other anthropometric parameters are significantly (*p* < 0.01) correlated with rowing ergometer performance (4). A very strong correlation (*R*^2 ^= 0.83; variance: 77%; SEE: 4.71) between multiple prediction equation based on anthropometric parameters (sitting height, lean body mass, body height, thigh girth, body weight, leg length and arm length) and 2000 m ergometer performance may be explained by the strong relationship between morphology and rowing performance (4). Despite some particular differences between ergometer and on-water performance, the 2000 m time trial on the rowing ergometer has become an important selection tool for national rowing organizations (5). When comparing on-water to simulated rowing methodologies only slight discrepancies in physiological responses exist (6). The most able rowers could be distinguished by their stature, skeletal robustness, and muscular development (7). According to De Larochelambart et al. (8) the fastest in single scull, in men and women, are tall and robust (in sculling disciplines each athlete operates two oars, unlike the sweep disciplines, where each rower operates only one larger oar). However, we must distinguish heavyweight and lightweight categories. According to the rules (9), a lightweight athlete competing in a single scull cannot exceed maximum weight limit of 72.5 kg for men, and of 59 kg for women. In all remaining crew disciplines (multiple athletes in a single boat), the average weight of the crew must not exceed 70 kg, and 57 kg respectively while at the same time no single male athlete can exceed 72.5 kg and 59 kg respectively for women. Weighing of athletes in rowing takes place 60–120 min prior the start of their first race of the day. Based on all of the above, it can be concluded that the long-term morphology monitoring of high-performance athletes is very important. Precisely identifying these morphological factors associated with performance, can assist the exercise scientist and coaches in selecting and developing talented athletes (10). There is, however, a lack of anthropometric data collected at either World Cups, World Championship or Olympic Games level to facilitate this talent identification approach. The aims of this study were to describe and compare the morphology of male and female heavyweight and lightweight rowers. Together with anthropometric parameters, strength is also a very important performance determinant in rowing (4, 11). Therefore, the rowers were also tested with a simple functional hand-grip test to determine strength ability. Unfortunately, this is usually the only simple functional test that rowers are willing to perform in such a close time to an important race. A unique opportunity to collect anthropometric and strength characteristics of elite rowers was presented during 2022 World Championship (18.–25. September, Račice, Czech Republic). With the official support of organizers and FISA, over 20 anthropometric measurements, body composition and hand-grip data were obtained from 68 competitors. This study should provide a better understanding of the body morphology of elite rowers, since the measurements were acquired from an elite performance group sample just one day prior to the start of the rowing world´s most important event of the year.

## Materials and methods

### Participants

Altogether 68 competitors of the World Rowing Championship 2022 in Račice (Czech Republic) were measured with a battery of anthropometric tests, segmental bioimpedance analysis and hand-grip strength test. Of 46 male competitors, 15 competed in the lightweight category (LWM) and 31 in the heavyweight category (HWM). Of 22 female athletes, 6 competed in the lightweight category (LWW) and 16 in the heavyweight category (HWW). Coxswains were not included.

Athletes were contacted and invited to take a part in this study through team officials and prospects distributed over the regatta venue. Only athletes competing during World Championship could take a part in this study. Specific rules for measurements were established. All participants read and signed informed a consent form before measurement. The study was approved by The Ethics committee at the Faculty of Physical Education and Sport, Charles University in Prague, Czech Republic.

### Data collection

The measurement took place over 2 days, just 1 day before the first World Championship race day (between 9 am and 5 pm) in regatta warm-up and boat preparation area. In order to collect higher amount of data (allow for more athletes to participate) the same procedure was run both days. To eliminate inter-rater variability, all measurements were conducted by experienced examiners from the Faculty of Physical Education and Sport. Each individual examination lasted approximately 20 min. Before the measurement, the athletes answered question about the boat category and the sport age, which was defined as the period of systematic rowing training.

### Anthropometric measurements

In the data collection of anthropometric parameters, standard methods were followed and licensed anthropometric instruments were used. Anthropometric measurements were carried out in accordance with standard anthropometric techniques recommended by Norton & Olds (12). Skinfold measurements were taken with a Harpenden skinfold caliper at the following sites: triceps, subscapular, suprailiac, thigh and calf. All unilateral measurements were performed on the right side of the body. Somatotypes were calculated according to Carter & Heath (13).

### Body composition

Body composition including body fat contribution was evaluated using the multifrequency device Tanita MC-980 MA (https://tanita.eu). Participants were asked not to eat for 2 h and drink 1 h before the measurement. Testing was performed in underwear only in a standing position with arms extended down.

### Grip strength

Handgrip isometric strength was assessed with a conventional dynamometer (Takei TKKK 5401, Takei Scientific Instruments, Tokyo, Japan). In a sitting position, the rowers grasped the hand dynamometer with an elbow in full extension, arm near the body, and gradually applied maximal pressure for at least 2 s. First, three trials with right arm and then three trials with left arm were examined. The best of three consecutive trials was considered for data analysis. A 30-s recovery was allowed between trials. While applying the grip force, the stretched hand was not allowed to touch any part of the body. The adjustable part of the handle was set to reach the first phalanx of the ring finger.

### Data analysis

From basic descriptive statistics mean and standard deviation is used. To find out the differences between the groups the independent student's *T*-test is used. Statistical significance was set at *p* < 0.05. Cohen's d was used to find practical differences. All statistical calculations were performed using IBM SPSS for Windows (version 24, Chicago, Il., USA). Effect sizes were classified as trivial (0–0.2), small (0.2–0.6), moderate (0.6–1.2), large (1.2–2.0) and very large (>2.0) (14). The radar-graphs with markers has been calculated for LWM and LWW as 10 − (0.5 × d), for HWM and HWW as 10 + (0.5 × d).

## Results

Table 1 shows the comparison between male and female heavyweight and lightweight rowers. Between HWM and LWM rowers there were a statistically and practically significant differences in all monitored aspects except the sport age, sitting height/body height ratio and arm span/body height ratio. Between heavyweight and lightweight female rowers there were also statistically and practically significant differences in all monitored aspects except the identical indicators as in men.

**Table 1:** Morphology, intersex and inter category differences of the 2022 world championship rowers.

Variable	Male rowers (*n *= 46)					Female rowers (*n *= 22)				
	Heavy weight (*n *= 31)	Light weight (*n *= 15)	Difference			Heavy weight (*n *= 16)	Light weight (*n *= 6)	Difference		
			*p*	*d*	%			*P*	*d*	%
Age (years)	25.6 ± 5.5	28.8 ± 6	0.04	0.56	11.1	23.6 ± 3	22.5 ± 1.4	0.19	0.42	4.9
Sport age (years)	11.3 ± 5.5	13.1 ± 6.4	0.16	0.31	13.7	9.9 ± 4.0	11.5 ± 2.0	0.19	0.43	13.9
Body mass (kg)	91 ± 8.8	72.3 ± 1.2	0.00	2.56	25.9	76.4 ± 5.5	59 ± 3.1	0.00	3.47	29.5
Height (cm)	189.9 ± 6.4	181.1 ± 4.3	0.00	1.51	4.9	178 ± 4.6	170.4 ± 3.8	0.00	1.72	4.5
Body mass index (kg/m^2^)	25 ± 2	22.1 ± 1.1	0.00	1.77	14.0	24.1 ± 1.7	19.9 ± 0.4	0.00	2.85	21.1
Sitting height (cm)	90.4 ± 3.6	86.6 ± 2.6	0.00	1.17	4.4	85.2 ± 3.1	82.1 ± 2.5	0.01	1.06	3.8
Arm span (cm)	192.6 ± 6.5	182.9 ± 5.8	0.00	1.53	5.3	178.8 ± 5.2	172.1 ± 2.7	0.00	1.44	3.9
Sitting height/body height (%)	47.6 ± 1.3	47.8 ± 1.3	0.3	0.14	0.4	47.9 ± 1.3	48.2 ± 0.9	0.3	0.25	0.6
Arm span/body height (%)	101.4 ± 2.5	101.0 ± 2.1	0.28	0.18	0.4	100.4 ± 2.3	102.0 ± 3.1	0.31	0.63	1.6
Shoulder breadth (cm)	46.7 ± 2.8	44.4 ± 2.6	0.00	0.86	5.2	43 ± 1.9	41.1 ± 2.2	0.03	0.93	4.6
Humerus breadth (cm)	7.6 ± 0.4	7.2 ± 0.5	0.00	1.09	5.6	6.4 ± 0.8	6.3 ± 0.3	0.36	0.17	1.6
Femur breadth (cm)	10.9 ± 0.7	10.1 ± 0.5	0.00	1.21	7.9	10.1 ± 0.7	9.3 ± 0.2	0.00	1.47	8.6
Forearm girth (cm)	30.1 ± 1.4	27.9 ± 1.3	0.00	1.62	7.9	26.3 ± 1	24.4 ± 0.8	0.00	2.15	7.8
Flexed arm girth (cm)	36.6 ± 2.1	32.6 ± 2.1	0.00	1.93	12.3	31.6 ± 1.5	27.7 ± 1.1	0.00	2.79	14.1
Chest girth (cm)	98.2 ± 5	91.7 ± 2.7	0.00	1.46	7.1	81.4 ± 6.4	75.5 ± 3.7	0.16	1.09	7.8
Thigh girth (cm)	57.8 ± 3.9	51.5 ± 2.3	0.00	1.79	12.2	57.5 ± 3.5	52.1 ± 1.1	0.00	1.75	10.4
Calf girth (cm)	39 ± 2.1	35.3 ± 1.5	0.00	1.9	10.5	37.8 ± 1.7	33.8 ± 0.9	0.00	2.55	11.8
Sum of 5 skinfolds (mm)	35.9 ± 7	26 ± 3.8	0.00	1.61	38.1	50.7 ± 12.4	36.1 ± 12.1	0.01	1.19	40.4
Body fat (%)	12.9 ± 4.1	8.3 ± 3.2	0.00	1.19	55.4	23 ± 3.5	15 ± 3.1	0.00	2.37	53.3
Endomorphy	2.2 ± 0.5	1.5 ± 0.7	0.00	1.15	46.7	3.1 ± 0.8	2.0 ± 0.8	0.00	1.38	55
Mezoporphy	5.6 ± 1.0	4.6 ± 1.2	0.02	0.90	21.7	4.4 ± 0.9	3.7 ± 0.5	0.00	0.96	18.9
Ectomorphy	2.3 ± 0.8	3.2 ± 0.7	0.00	1.19	28.1	2.1 ± 0.7	3.4 ± 0.6	0.00	1.99	38.2
Hand-grip right hand (kgf)	59 ± 8.9	51.6 ± 6.8	0.02	0.89	14.3	40.8 ± 6.7	33.1 ± 5.3	0.00	1.22	23.3
Hand-grip right hand relativized (kgf.kg^−1^)	0.7 ± 0.1	0.7 ± 0.1	0.04	0.57	0	0.5 ± 0.1	0.6 ± 0.1	0.2	0.41	16.7
Hand-grip left hand (kgf)	58.2 ± 8.3	50.1 ± 6.6	0.00	1.04	16.2	40.5 ± 7.6	32.2 ± 4.4	0.00	1.19	25.8
Hand-grip left hand relativized (kgf.kg^−1^)	0.6 ± 0.1	0.7 ± 0.1	0.06	0.49	14.3	0.5 ± 0.1	0.6 ± 0.1	0.3	0.25	16.7
TBW (%)	62.3 ± 3	65.4 ± 2.8	0.00	1.06	4.7	56.7 ± 2.6	59.9 ± 2.5	0.00	1.26	5.3
ECW/TBW (%)	33.2 ± 1.2	35 ± 1.2	0.00	1.48	5.1	35 ± 2.4	36.8 ± 0.9	0.00	0.85	4.9

**Figure 1:**
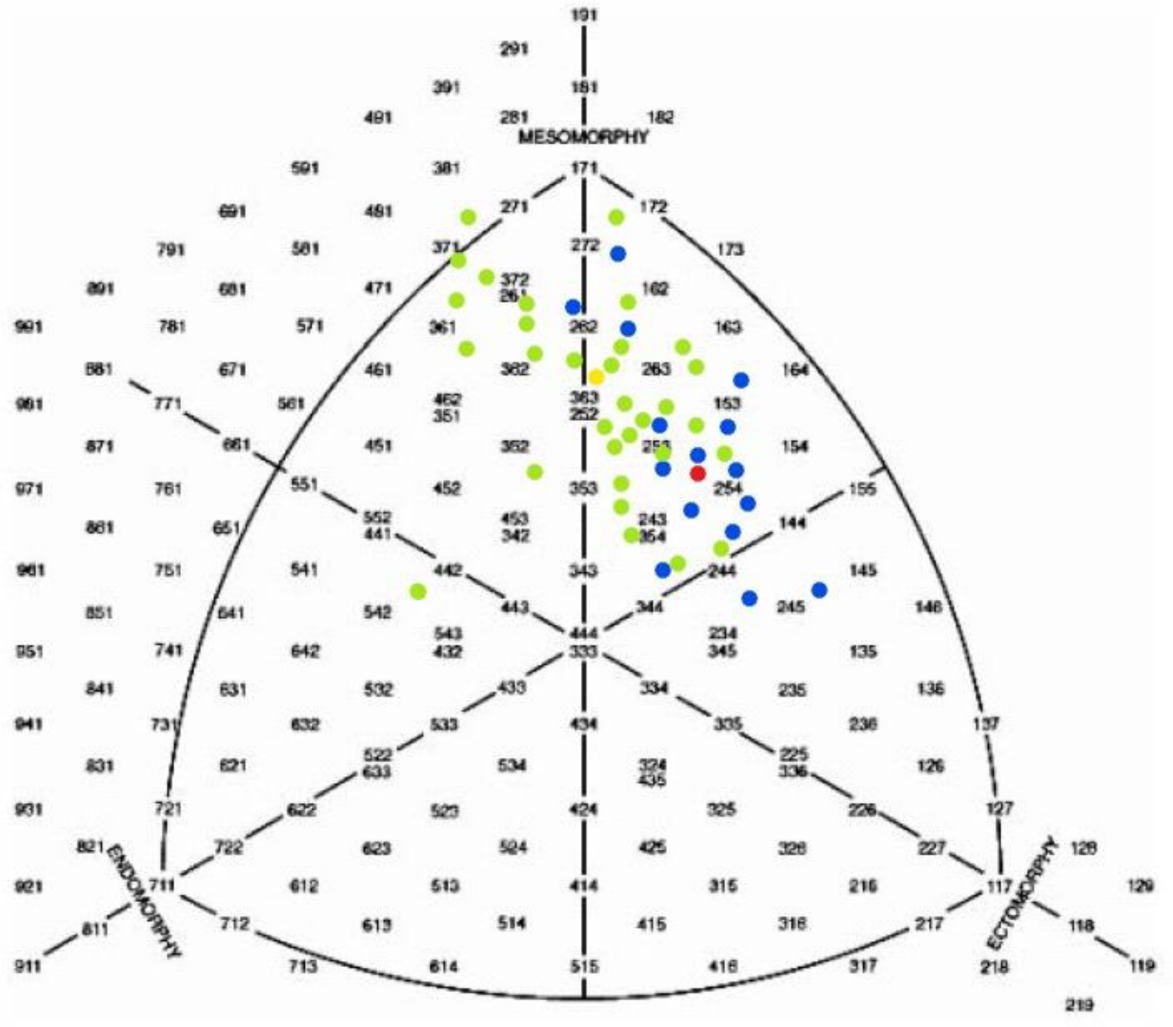
Somatograph of male rowers: ●individual somatotype of heavyweight rowers; ●average somatotype of heavyweight rowers (2.2–5.6–2.3); ●individual somatotype of lightweight rowers; ●average somatotype of lightweight rowers (1.5–4.6–2.3).

**Figure 2:**
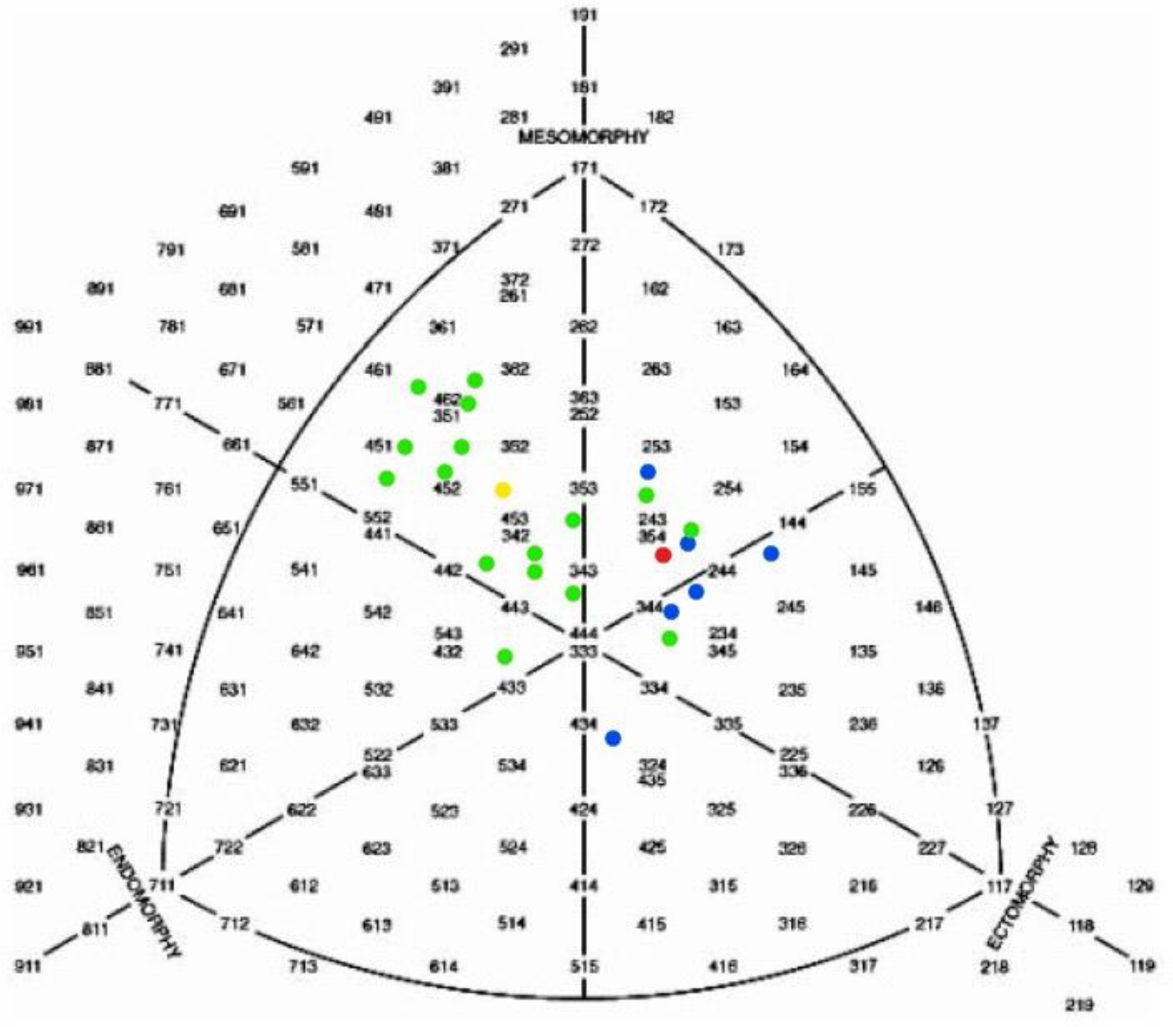
Somatograph of female rowers: ●individual somatotype of heavyweight rowers; ●average somatotype of heavyweight rowers (3.1–4.4–2.1); ●individual somatotype of lightweight rowers; ●average somatotype of lightweight rowers (2.0–3.7–3.4).

In Figures 1, 2 somatotypes of all participants are shown. There are significant differences between HWM and LWM in all somatotype dimensions. The same applies for HWW and LWW.

**Figure 3:**
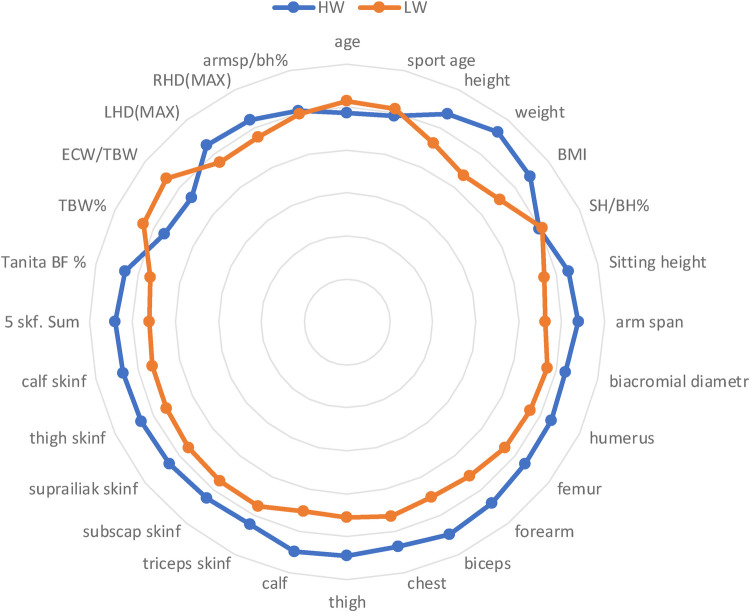
Radar graph with markers to present intergroup differences in males.

**Figure 4:**
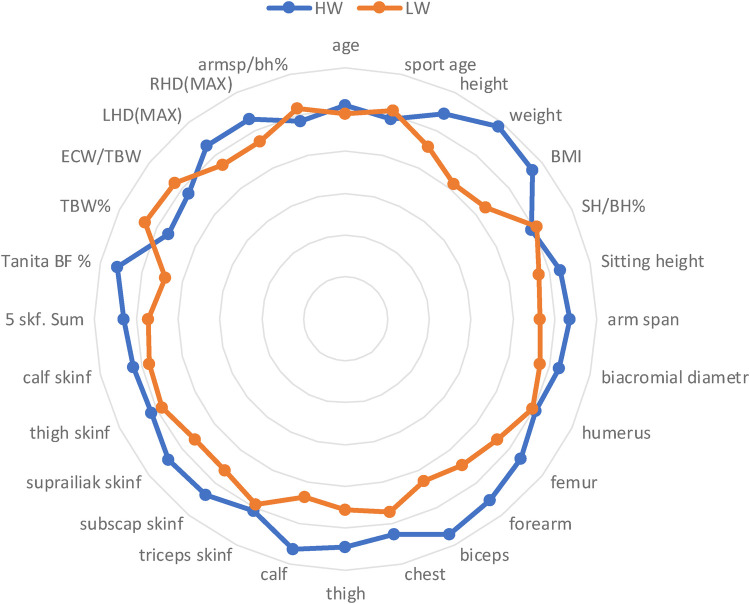
Radar graph with markers to present intergroup differences in females.

Radar graphs (Figures 3, 4) present graphically differences between HW and LW categories. Body constitution, composition and strength ability of HWM and HWW differ radically from LWM and LWW.

**Figure 5:**
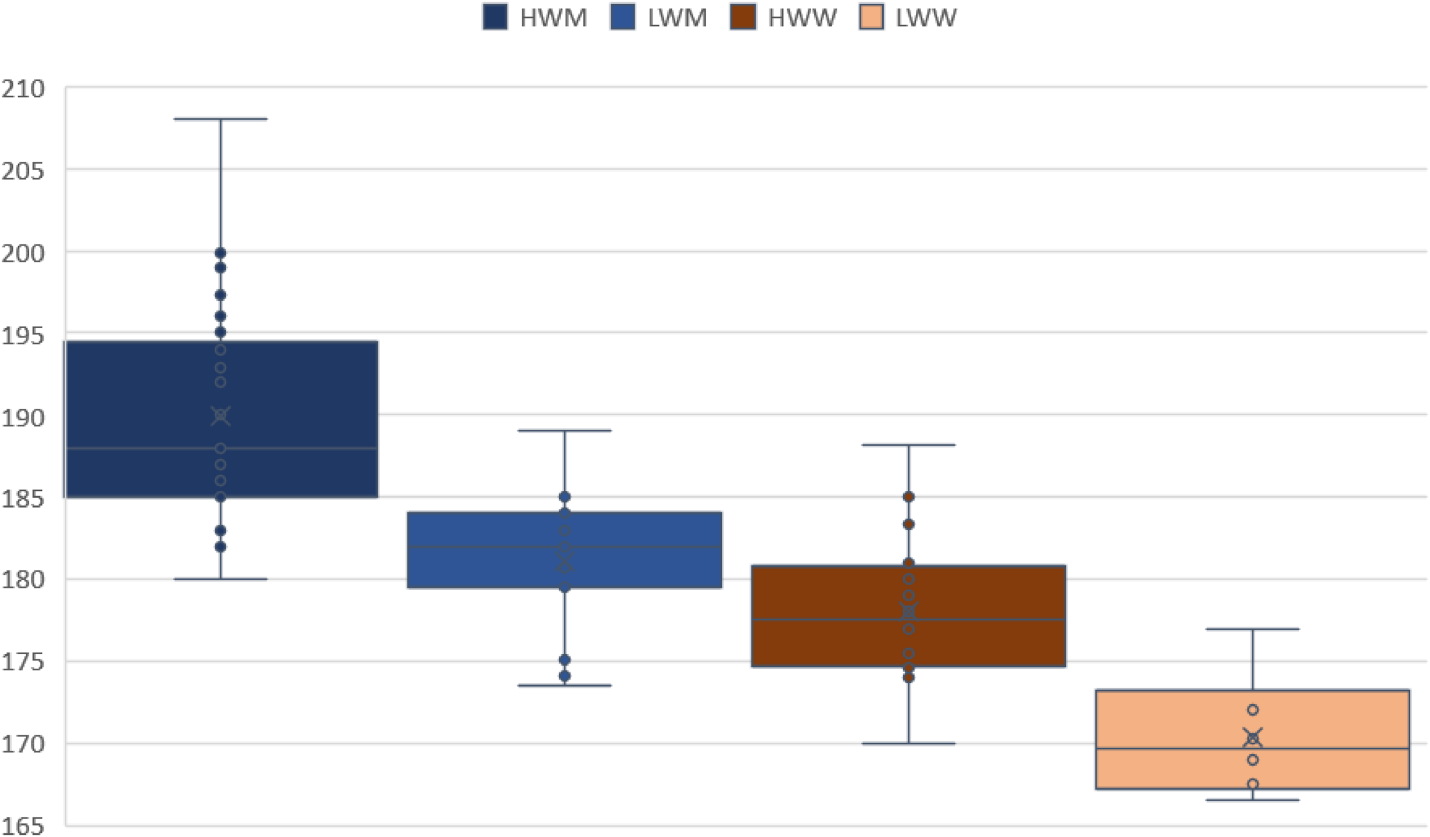
Differences in body height in male and female rowers.

**Figure 6:**
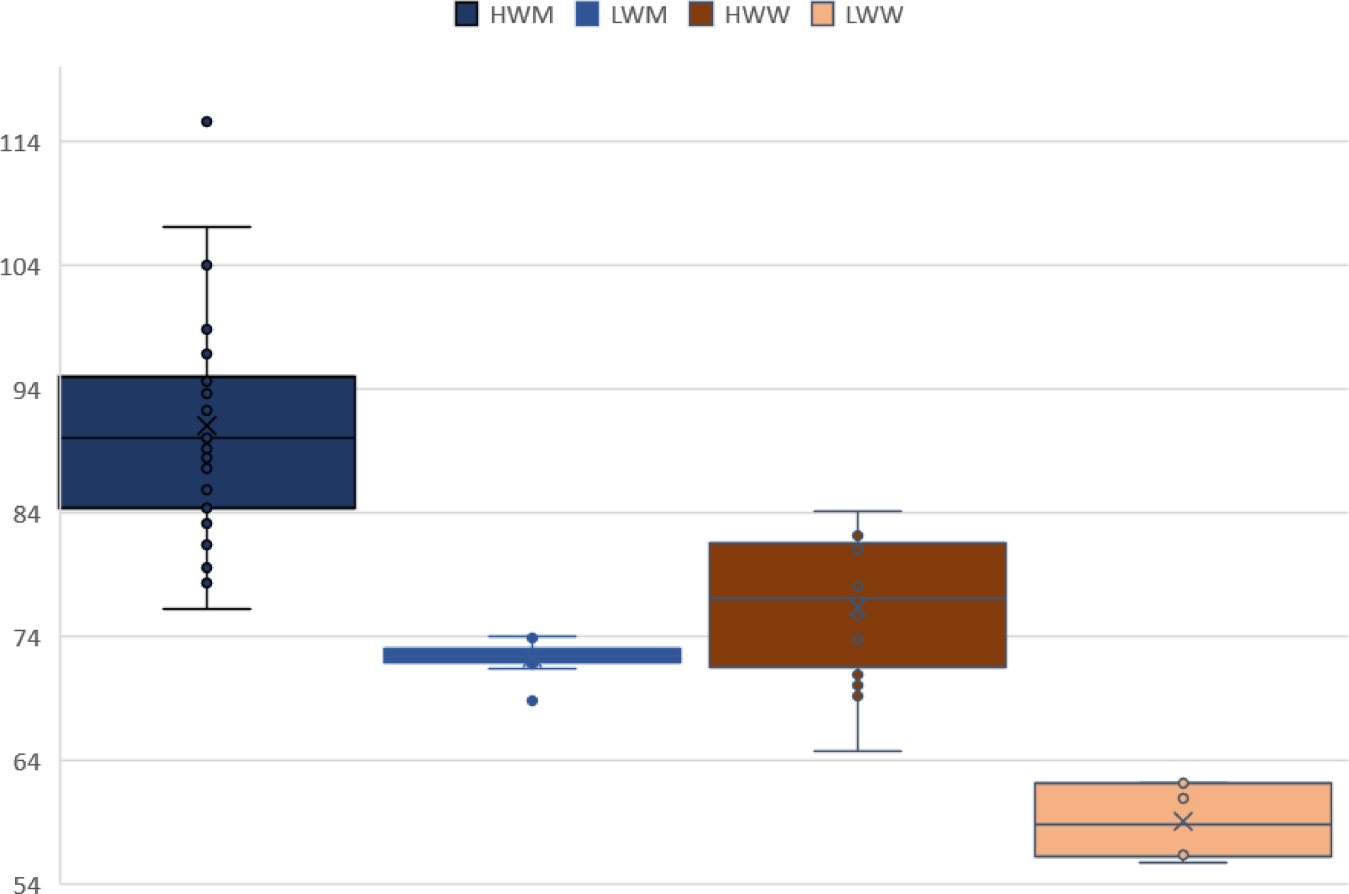
Differences in body weight in male and female rowers.

In Figures 5, 6 can be observed a very wide range of body height and weight values for the HW categories. Narrower range of values is evident in LW categories, especially in the body weight. Male and female rowers are more homogeneous in terms of body morphology.

## Discussion

### Male rowers

According to Kerr et al. (15) the average weight and height of HW male rowers participated at 2000 Sydney Olympic was 94.3 ± 5.9 kg and 193.3 ± 4.9 cm. But the best athletes (top 7 places) were even higher (194.1 ± 4.4 vs. 191.5 ± 5.7 cm), heavier (95.3 ± 5.4 vs. 91.9 ± 6.4) and had more muscular upper body (flexed arm girth: 36.8 ± 1.7 vs. 35.9 ± 1.9; forearm girth: 31.2 ± 1.3 vs. 30.5 ± 1.3; chest girth: 109.2 ± 4.2 vs. 107.5 ± 4.7 cm). The elite male junior rowers were tall (187.4 ± 5.8 cm) and heavy (82.2 ± 7.4 kg), with larger length, breadth, and girth dimensions than the reference population of boys of the same chronological age (3). Bourgois et al. (3) found significant (*p* < 0.01) differences between finalists and non-finalists in 1997 World Junior Rowing Championship. Finalists were heavier and had higher values for length, breadth, and girth dimensions. Within the older juniors, internationally ranked rowers had significantly greater body height (+5.9 cm), body mass (+6.1 kg), sitting height (+2.7 cm), arm span (+7.9 cm) and limb length (+3.7 cm). They also rowed 2000 m significantly faster, had higher values of power (+58.3 W), relative power (+0.41 W/kg), maximal speed (+0.18 m/s), and force (+163.2 N) (16). HWM 2022 World Championship participants in this study were consistent with previous studies very tall, heavy, muscular, with relatively long limbs and short torsos. The mean somatotype of HW rowers in this study (2.2–5.6–2.3) was more mesomorphic than the mean somatotype established in the study of Das et al. (17) (1.9–4.1–3.1) and even in a study tracking HW rowers at the 2000 Sydney Olympics (1.9–5.0–2.5) (15). Majmudar et al. (18) found that rowers with higher mesomorphy took less time to complete 2 km rowing. Thus, identifying the mesomorphic component which represents muscularity is important for the evaluation of a rower.

It is known that a LW meńs crew shall have an average weight not exceeding 70 kg. No individual LW male rower may weigh more than 72.5 kg (19). HW rowers differ significantly from LW rowers in all aspects of body morphology. HW rowers are taller (+8.8 cm, +4.9%), heavier (+18.7, +25.9%), more muscular (flexed arm girth: +4 cm, +12.2%; forearm girth: +2.2 cm, +7.9%; chest girth: +6.5 cm, +7.1%; thigh girth: +6.3 cm, +12.2%, calf girth: +3.7 cm, +10.5%), more mesomorphic (+1 point, +21.7%). HW rowers had higher body fat percentage (12.9 vs. 8.3%). Body fat was significantly higher in HWM compared to LWM (+24.4%) in the study of Das et al. (17). Large significant (*p* < 0.01) difference in body fat was also found by Kerr et al. (15) in sum of 8 skinfolds (65.3 vs. 44.7 mm). However, according to Kerr et al. (2007) there was no significant difference in the sum of skinfolds between finalists and non-finalists. Therefore, it seems that keeping the body fat low is important in lightweight categories, whereas for HW rowers higher body fat is not limiting. This paper confirms the conclusion of Kerr et al. (15) from 2000 Sydney Olympic Games that HWM and LWM rowers are completely different in their body morphology.

The morphology difference between HW and LW male rowers is also reflected in strength abilities, which in this study were monitored only through handgrip. HWM had a bigger handgrip strength (59 ± 8.9) than LWM (51.6 ± 6.8) significantly (*p* = 0.02; *d* = 0.89) by 14.3%, confirming the findings of Das et al. (17) who observed very similar handgrip strength in LWM (51.1 ± 1.9) and HWM (56.2 ± 4.5). This difference in absolute values is to a high degree determined by the difference in the constitution of the rowers themselves (20). The differences in relative strength between HW and LW were not significant. According to Cronin et al. (21), in some sports where hand grip strength is believed to play a role in performance had still minimal research attention, such as paddling sports (e.g., kayaking, rowing, and canoeing), hockey (ice and field), basketball, volleyball, riding (horses, bulls, bikes, and motorcycles), and driving (race cars). The most similar to rowing are kayaking and canoeing. The hand-grip strength of the HW is similar to that of an international level canoe slalom paddlers (57.0 kgf) presented by Busta, Coufalová & Cochrane (22) or Czech national team canoe slalom paddlers (23). Lower hand-grip strength level of LW rowers is more similar to university level of canoe sprint paddlers (50.0 kgf) of Japan (Hamano et al., 2015).

### Female rowers

A LW women's crew shall have an average weight not exceeding 57 kg. No individual LW female rower may weight more than 59 kg (19). Within this research it can be argued that female HW rowers are in many anthropometric aspects more similar to their male counterparts than to female rowers in the LW category. The physical characteristics of elite female lightweight rowers differ radically from those of heavyweight. In some anthropometric aspects (BMI, thigh girth, calf girth), female rowers are even more similar to HWM than to LWM rowers. Also according to Bourgois et al. (3) the group of elite female junior rowers were, on average, 6.7 cm taller and 11.9 kg heavier than elite female LW rowers. Similar to men, it can be concluded that the morphology of HW rowers is completely different from that of LW category. Similar to men, in our research HWW had significantly (*p* = 0.00; *d* = 1.19) more body fat than LW women by 8%. According to Bourgois et al. (3) within the group of elite female rowers, differences exist between finalists and non-finalists in length, breadth and girth dimensions and body mass. Kerr et al. (15) found a significant difference in female rower finalists and non-finalists just in the sum of skinfold thickness and the endomorphic component of the somatotype. The sum of 8 skinfolds was lower in the best rowers compared to the rest by 19.7 mm (82.1 ± 23.2 vs. 99.8 ± 20.4 mm). Excessive body fat can have a more negative effect on performance in women than in men, as they may not achieve the recommended relative strength levels (11) due to their higher weight. This morphological difference also determines the significant difference in handgrip strength (*p* = 0.00; *d* = 1.22) by 23%. As in men, the relativized handgrip performances were not evaluated as significant in women. In the study of Almeida-Neto et al. (24), female rowers achieved the highest (*p* < 0.0001) values of all the sports disciplines studied (swimming, soccer, jiu jitsu, tennis and volleyball). According to Almeida-Neto et al. (24) girls aged 13 to 16 years were able to perform a handgrip strength almost 40 kgf. This value is higher than what was found for LW female rowers in this study. However, according to Almeido-Neto et al. (2020) maturity status is related with strength development. This explain that the young girls (body weight: 65.1 ± 16.7 kg; body height: 167.7 ± 9.6 cm) have reached higher handgrip values.

## Practical applications

From a practical point of view, this research can be used to determine what type of somatotype should be recruited or selected for open category and what type for lightweight category in male and female rowing. Our research confirms the fact that above-average height, mass and other anthropometric parameters such as breadths, widths and girths should be prioritized in early selection, because HW and LW rowers have completely different morphology. This fact should be considered from the beginning and during the athletés career to select the appropriate weight class and boat category.

## Data Availability

The original contributions presented in the study are included in the article/Supplementary Material, further inquiries can be directed to the corresponding author/s.

## References

[1] Shephard RJ. Science and medicine of rowing: a review. J Sports Sci. (1998) 16(7):603–20. 10.1080/026404198366416

[2] Winkert K Steinacker JM Machus K Dreyhaupt J Treff G. Anthropometric profiles are associated with long-term career attainment in elite junior rowers: a retrospective analysis covering 23 years. Eur J Sport Sci. (2019) 19(2):208–16. 10.1080/17461391.2018.1497089 30062920

[3] Bourgois J. Anthropometric characteristics of elite male junior rowers. Br J Sports Med. (2000) 34(3):213–6. 10.1136/bjsm.34.3.213 10854024 PMC1763275

[4] Mikulić P Ruzić L Oreb G. What distinguishes the Olympic level heavyweight rowers from other internationally successful rowers? Coll Antropol. (2007) 31(3):811–6. PMID: 18041393

[5] Akça F. Prediction of rowing ergometer performance from functional anaerobic power, strength and anthropometric components. J Hum Kinet. (2014) 41(1):133–42. 10.2478/hukin-2014-0041 25114740 PMC4120446

[6] Smith TB Hopkins WG. Measures of rowing performance. Sports Med. (2012) 42(4):343–58. 10.2165/11597230-000000000-00000 22401296

[7] Vogler AJ Rice AJ Gore CJ. Physiological responses to ergometer and on-water incremental rowing tests. Int J Sports Physiol Perform. (2010) 5(3):342–58. 10.1123/ijspp.5.3.342 20861524

[8] Piotrowski J Sklad M Krawczyk B Majle B. Somatic indices of junior rowers as related to their athletic experience. Biol Sport. (1992) 9:118–25.

[9] De Larochelambert Q Del Vecchio S Leroy A Duncombe S Toussaint JF Sedeaud A. Body and boat: significance of morphology on elite rowing performance. Front Sports Act Living. (2020) 2:597676. 10.3389/fspor.2020.597676 33345179 PMC7739618

[10] Ackland T. Built for success: homogeneity in elite athlete morphology. In: Marfell-Jones M Stewart A Olds T, editors. Kinanthropmery IX. UK: Routledge Vol. 1. (2006). p. 29–38, (Proceedings of the 9th International Conference of the International Society for the Advancement of Kinanthropometry).

[11] McNeely E Sandler D Bamel S. Strength and power goals for competitive rowers. Strength Cond J. (2005) 27(3):10–5. 10.1519/00126548-200506000-00001

[12] Norton K Olds T, Australian Sports Commission. Anthropometrica: A textbook of body measurement for sports and health courses. Sydney, Australia: UNSW Press (1996).

[13] Carter JEL Heath BH. Somatotyping, development and applications Vol. 1. Cambridge: Cambridge University Press (1990). publ. (Cambridge studies in biological anthropology).

[14] Hopkins WG. A Scale of Magnitudes for Effect Statistics, A New View of Statistics (2006). Available at: https://www.sportsci.org/resource/stats/effectmag.html (Accessed January 18, 2023).

[15] Kerr DA Ross WD Norton K Hume P Ackland TR. Olympic Lightweight and open-class rowers possess distinctive physical and proportionality characteristics. J Sports Sci. (2007) 25(1):43–53. 10.1080/02640410600812179 17127580

[16] Alfőldi Z Borislawski K Ihasz F Soós I Podstawski R. Differences in the anthropometric and physiological profiles of Hungarian male rowers of Various age categories, rankings and career lengths: selection problems. Front Physiol. (2021) 12:747781. 10.3389/fphys.2021.747781 34721071 PMC8548758

[17] Das A Mandal M Majumdar P Syamar AK. Morpho-physiological profile and 2 K performance of Indian elite rowers. J Phys Educ Sport. (2019) 19:1630–5. 10.7752/jpes.2019.03236

[18] Majmudar P Das A Mandal M. Physical and strength variables as a predictor of 2000 m rowing ergometer performance in elite rowers. J Phys Educ Sport. (2017) 17(04):2502–7. 10.7752/jpes.2017.04281

[19] FISA. FISA Rulebook. Lausanne: FISA (2022). Available at: https://worldrowing.com/2014/05/22/rule-book/

[20] Jürimäe T Hurbo T Jürimäe J. Relationship of handgrip strength with anthropometric and body composition variables in prepubertal children. HOMO. (2009) 60(3):225–38. 10.1016/j.jchb.2008.05.004 18996520

[21] Cronin J Lawton T Harris N Kilding A McMaster DT. A brief review of handgrip strength and sport performance. J Strength Cond Res. (2017) 31(11):3187–217. 10.1519/JSC.0000000000002149 28820854

[22] Busta J Coufalová K Cochrane DJ. Strength and stregth-related anthropometric parameters of the international level canoe slalom male paddlers. Inter J Morphol. (2022) 40(3):579–83. 10.4067/S0717-95022022000300579

[23] Busta J Tufano JJ Suchý J Bílý M. Anthropometric, physiological and performance profiles of elite and sub-elite canoe slalom athletes. J Outdoor Act. (2019) 12(1/2018):53–61. 10.14712/23366052.2018.5

[24] Almeida-Neto PF Matos DG Baxter-Jones ADG Batista GR Pinto VCM Dantas M The effectiveness of biological maturation and lean mass in relation to muscle strength performance in elite young athletes. Sustainability. (2020) 12(17):6696. 10.3390/su12176696

